# What Is "Immediate Personal Supervision"?

**Published:** 1899-07-01

**Authors:** 


### Editor's Letter-Box.

[Our Correspondents are reminded that prolixity is a great bar to publication, and that brevity of style and conciseness of statement greatly facilitate early insertion.]

### WHAT IS "IMMEDIATE PERSONAL SUPERVISION"?

"Medical Practitioner" writes: With reference to your annotation in The Hospital some little time ago on the subject of "Hospitals Without House Surgeons," I wish to ask, in view of the recent resolution of the General Medical Council with regard to the employment of unqualified assistants by medical practitioners, if the institute referred to hereafter and its medical and surgical staffs are acting strictly within the letter and spirit of that resolution or not, and whether or not a general medical practitioner is at liberty to act in a similar manner without incurring the risk of being judged guilty of infamous conduct in a professional way. At this stage I will remark that I have nothing to say against the charity in question. I only wish to inquire if the system of carrying it on is legal or otherwise, and if a corporation such as is pointed out in this letter can do what a medical practitioner in his private capacity is prohibited from carrying out. All the statements I give are in existence, and I can vouch for their accuracy. Without giving the name, the town from which I write has a charity lying-in institute. The latter has at varying distances of from half a mile to a mile or more two at least branch establishments. All these are each manned, if I may so express it, by one or two trained or qualified midwives with numerous probationers. The parent institute has in addition a resident qualified and registered house surgeon and a medical and surgical visiting staff. I should mention, perhaps, that the branch establishments are only residences for the convenience of the midwives in the different localities, and of the poor at whose homes the midwives attend. The branch establishments have no house surgeons, being under the charge of the staffs of the parent institute, from which also midwives go out to attend labour cases in the same way as midwives from the branches do. The work of all these establishments is carried out as follows: When "a call" comes in a midwife, accompanied by one, two, or three (as the case may be) probationers, go out to the case. They carry through the confinement, if possible, without the aid or "immediate personal supervision" of either the house surgeon or any other member of the visiting staff—only in case of difficulty or danger is one of the latter sent for. The house surgeon is afterwards made aware of the parturition, and he is expected to visit the case once only within the ten days following, provided everything goes on well. Of course if anything goes wrong, such as puerperal fever, &c., he takes the charge and the responsibility of the patient, and, to my mind, the "covering" of unqualified persons. The midwives during the lying-in period attend and treat the patient if she goes on well until all right without any supervision whatever, except that which I have mentioned as fulfilled by the house surgeon. Taking these facts as they stand, I would inquire whether the employment of midwives as here depicted, where the "immediate personal supervision" of a registered medical practitioner is dispensed with, is legitimate or not? In my opinion it is illegal, and I consider the registered medical practitioners who are privy to and who sanction the substitution of their services to unqualified persons, and who knowingly countenance, assist, and co-operate in the employment of unqualified persons to attend and treat patients in respect of matters requiring professional discretion and skill in the manner I have pointed out here, are guilty of "covering" and of employing unqualified assistants and are liable to the censure of the General Medical Council. I understand that the General Medical Council have lately stated that it would not consider a man was guilty of infamous conduct who attended midwifery with a midwife, *i.e.*, when she got into difficulties. This leaves their resolution nothing clearer, and I think was quite an unnecessary explanation. For when a midwife undertakes a case on her own responsibility, and getting into difficulties sends for a registered practitioner, who goes to her help, how could he be judged guilty of "infamous conduct," seeing that he is the employé and not the employer of the unqualified person? There is no law, that I am aware of, against an unqualified person employing a qualified person to help him or her in a case of a difficult confinement, and as long as the qualified person does not do anything to make people believe that the unqualified person is qualified the qualified person cannot, if I understand aright, be touched by the council. Again, few practitioners care to "follow" midwives, unless the patients are poor, when they usually give their services gratuitously; but they (the practitioners) in no sense employ the midwives by so "following"; therefore, I consider the statement of the General Medical Council quite a superfluous quantity, as no one could seriously think that helping a midwife out of a difficulty when he did not employ her for the case could fall within the resolution referred to. In conclusion, I would inquire what the position of a medical practitioner would be if he took houses in different parts of a town and put them in charge of midwives to carry out his work in a similar way as the institute referred to before does. Surely the one is as justified as the other in acting so. In parish and Poor Law appointments I believe a like state of affairs exists as in the institute introduced here. Why is this not put a stop to by the General Medical Council? What is sauce for the goose is sauce for the gander, and I trust you will expose the anomaly.

